# How Shall Reciprocity in Licensure Be Established?

**Published:** 1900-09

**Authors:** 


A Monthly Review of Medicine and Surgery.

EDITOR:

WILLIAM WARREN POTTER, M. D.

All communications, whether of a literary or business nature, books for review and exchanges should be addressed to the editor: 284 FRANKLIN STREET, BUFFALO, N. Y.

VOL. XL.—LVI. SEPTEMBER, 1900. NO. 2.

### HOW SHALL RECIPROCITY IN LICENSURE BE ESTABLISHED?

UNTIL a few years ago the opprobrium of medical education in the United States was the admission of candidates to the doctorate degree with inadequate preparation. American diplomas for this reason, with few exceptions, were denied recognition. A limited number of our colleges demanded a preliminary education for admission, and required a longer period of training, but the rule was little or no preliminary qualification and only two years in college, most of which was spent in attendance upon didactic lectures.

Every foreigner, however, who presented a diploma, no matter where or how obtained was admitted to practise unchallenged. Here was reciprocity with a very small "r;" it was a kind of reciprocity that didn't reciprocate.

Within the last few years a considerable number of states have established examinations for license, each regulating the methods as to preliminaries and training according to environment, political conditions, geographical situations, the liberal education of legislators, sentiment, and other factors entering into a solution of the question in the several commonwealths. While all have had a single object in view—namely, an improved and better educated medical profession, there has yet been lack of homogeneity in laws and regulations among the several states. As a consequence, standards have varied sufficiently to make it impossible for licentiates to obtain interstate indorsement of their credentials without injustice to those whose licenses were granted by states having highest standards. In other words, a physician holding a license from a state having the lesser standard, could not pass to a state having a greater standard without examination by the examining board representing the latter state. Thus, it is easy to understand why a state will not admit those to practise within its borders, who hold licenses obtained under systems less rigid than it applies to its own citizens. The state is zealous of its rights and guards the interests and privileges of its own citizens. Very properly so.

It is a mistake to hold examining boards responsible for these apparent incongruities. Each state fixes its own laws, regulates its own standards, and then appoints an examining board to execute them. The functions of the boards are administrative not creative. There has been much discussion in societies and journals as to the methods of establishing interstate reciprocity, but among it all we have seen nothing in print that presents the matter with greater clearness or intelligence than an article in the editorial columns of the New York *Medical Record*, issue of July 28, 1900, which we take pleasure in reproducing in its entirety, to-wit:

THE NEED OF UNIFORMITY IN THE STANDARDS OF MEDICAL EDUCATION.

During the current year attention has been called on several occasions in the *Medical Record* to the question of interstate reciprocity in medical licensing and the best means of bringing about this most desirable event has been touched upon. On February 10th in an article "Medical Education in the United States," the opinion was given that the National Confederation of State Medical Examining and Licensing Boards was the only body fitted by its composition to deal efficiently with the matter, and that to it must be left the solving of the problem, and the hope was then expressed that at its meeting at Atlantic City a discussion of the subject in all its bearings might tend to evolve order from the present decidedly chaotic condition of affairs.

We are glad to say that this hope has been realised to a considerable extent. The Confederation met in Atlantic City on June 4th, and the reciprocity difficulty was pretty thoroughly threshed out. Drs. William Warren Potter, of Buffalo; James A. Eagan, of Springfield, Ill.; E. B. Harvey, of Boston; J. N. McCormack, N. R. Coleman, of Columbus, Ohio, and others took part in the discussion. The agreement was unanimous as to the need of reform in the existing state medical laws, but the concensus of opinion was that any movement to this effect should be deliberate and well considered, and that nothing should be done hastily. The National Confederation appointed an interstate reciprocity committee consisting of the following members: Dr. Spurgeon, of Indiana, chairman; Dr. Amberg, of Detroit, secretary; Drs. Harvey, of Massachusetts; Korndoorfer, of Pennsylvania; and Swarts, of Rhode Island. The section on state medicine of the American Medical Association appointed an interstate reciprocity committee of three: Dr. Motler, of Washington, D.C., chairman; Drs. Amberg, of Detroit and McIntire, of Easton, Pa.

That interstate reciprocity is badly needed will be allowed by all, but that under the present conditions it is impossible is a fact just as indisputable. The first steps to be taken is the introduction, as far as is possible, of a uniform standard of medical education throughout the country. Mr. James Russell Parsons, the author of an excellent monograph on the subject, suggests as a uniform standard for admission to medical practice in all parts of the United States is at present impracticable, that instead of the adoption of a separate standard for almost every political division two, or at most three, standards should answer for all. One phase of the situation is both noteworthy and satisfactory; the members of the medical profession of this country are becoming more and more alive to the incongruity, not to say absurdity, of the existing methods of medical education in the various states. After all, the remedy lies in their own hands. If they determine that the standards shall be raised and placed upon a uniform plane, all obstacles will quickly melt away.

The whole question of reciprocity really depends upon a uniformity of educational methods to be adopted by the colleges. When this is done there will be no difficulty in establishing interstate indorsement. Until that desirable consummation is reached all attempts of reciprocity will prove futile. We have on more than one occasion presented this view of the question to our readers, and take pleasure in adding our full indorsement to the able and forceful review of the situation by our esteemed contemporary as above quoted.

